# Li^+^ Selective Podand-Type Fluoroionophore Based on a Diphenyl Sulfoxide Derivative Bearing Two Pyrene Groups

**DOI:** 10.3390/molecules16086844

**Published:** 2011-08-10

**Authors:** Yukihiro Nishimura, Tetsuo Takemura, Sadao Arai

**Affiliations:** 1 Department of Chemistry, Tokyo Medical University, 6-1-1 Shinjuku, Shinjuku-ku, Tokyo 160-8402, Japan; Email: s-arai@tokyo-med.ac.jp; 2 Department of Chemistry, Tokyo University of Science, 1-3 Kagurazaka, Shinjuku-ku, Tokyo 162-8601, Japan; Email: ttakemur@rs.kagu.tus.ac.jp

**Keywords:** fluoroionophore, podand, pyrene, fluorescence, lithium ion

## Abstract

New podand-type fluoroionophores having two pyrene moieties: 2,2´-bis(1-pyrenylacetyloxy)diphenyl sulfide (**3**), 2,2´-bis(1-pyrenylacetyloxy)diphenyl sulfoxide (**4**), and 2,2´-bis(1-pyrenylacetyloxy)diphenyl sulfone (**5**), have been synthesized by connecting two 1-pyrenecarbonylmethyl groups with the two hydroxy groups of 2,2´-dihydroxydiphenyl sulfide, sulfoxide, and sulfone, respectively. Their complexation behavior toward alkali metal ions was examined by fluorescence spectroscopy. Among these fluoroionophores, compound **4**, having a sulfinyl group, showed high selectivity toward Li^+^.

## 1. Introduction

Design and synthesis of fluoroionophores for metal ions is a vigorous research area [1,2,3,4,5,6]. These fluoroionophores have an ion recognition unit (such as a crown ether, calixarene or thiacalixarene) and a fluorophore unit (such as dansyl, anthracenyl, and pyrenyl groups) connected by appropriate linkers. When the ion recognition unit interacts with the target metal ions, chemical signals, such as absorbance or fluorescence intensities, are typically generated or attenuated in the fluorophore unit.

Among alkali metal ions a number of fluoroionophores for Na^+^ and K^+^ have been reported because these ions play particular roles in the regulation of many biological events. [7,8,9]. We have previously reported a *p*-*tert*-butylcalix[4]arene derivative which is connected with *p*-*tert*-butylcalix[4]arene and pyrene by a CH_2_CO linker [10]. This derivative shows an increase of pyrene monomer emission and a decrease of pyrene excimer emission with increased Na^+^ concentration. Subsequently, we have synthesized podand-type fluoroionophores, with two pyrene units **1** and **2 ** using 2,2´-dihydroxydiphenylmethane as a precursor of calix[4]arene and 2,2´-dihydroxydiphenyl ether, respectively, and examined the binding abilities toward alkali metal ions [11]. Although **1** has only slight affinity for alkali metal ions, **2** has good sensitivity for Na^+^ showing an increase of pyrene mononer emission and a decrease of pyrene excimer emission with increased Na^+^ ion. The oxygen atom between two phenyl groups in fluoroionophore **2** was found to play a crucial role in binding Na^+^. This observation led us to construct novel podand-type fluoroionophores in which the oxygen atom between the two phenyl groups in **2** is replaced by different types of atoms with binding abilities for alkali metal ions other than Na^+^.

**Figure 1:**
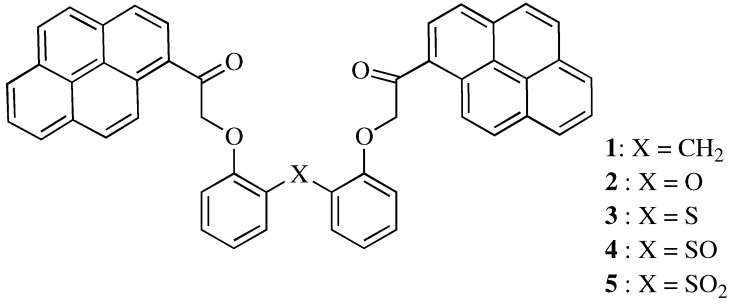
Fluoroionophores **1**, **2** and novel compounds **3**, **4**, and **5**.

Fluoroionophores for Li^+^ have been developed recently with regard to their medical and clinical applications for the treatment of manic-depressive psychosis [12,13,14]. However, relatively few studies concerning fluoroionophores for Li^+^ have been reported compared with those for Na^+^ and K^+^. Here, we report the synthesis of novel podand-type compounds **3**–**5** (Figure 1) having two pyrene units as a fluorophore unit and a sulfur atom, sulfinyl group, or sulfonyl group as a binding site. Binding studies of compounds **3**–**5** toward alkali metal ions (Li^+^, Na^+^, K^+^, Rb^+^, Cs^+^) have shown that compound **4**, which has a sulfinyl group, has a good binding ability for Li ^+^.

## 2. Results and Discussion

**Scheme 1:**
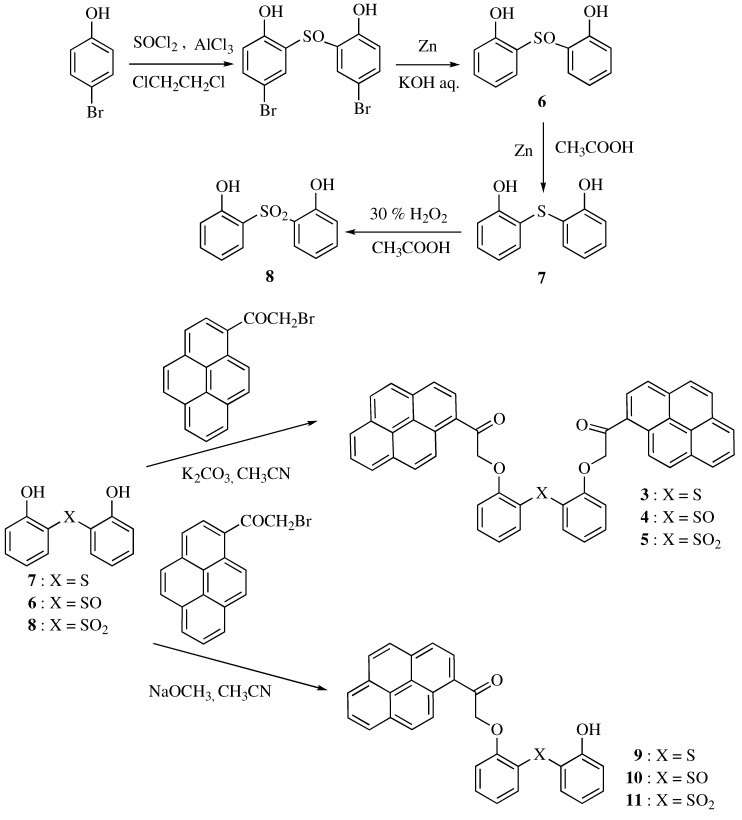
Synthetic routes of compounds **3**, **4**, and **5**, with two pyrene units, and **9**, **10**, and **11**, with one pyrene unit.

The fluoroionophores **3**–**5** were synthesized by connecting two 1-pyrenecarbonylmethyl groups with the two hydroxy groups of 2,2´-dihydroxydiphenyl sulfide, sulfoxide, or sulfone, respectively (Scheme 1). First, 2,2´-dihydroxydiphenyl sulfoxide **6**, sulfide **7**, and sulfone **8** were synthesized using 4-bromophenol as a starting material according to a reported procedure [15]. Then, compounds **3**, **4**, and **5,** with two pyrene units, were synthesized by the reaction of **7**, **6**, and **8**, respectively, with two equivalents of 1-(bromoacetyl)pyrene in the presence of potassium carbonate in acetonitrile. Furthermore, compounds **9**, **10**, and **11**, with one pyrene unit, were synthesized as reference compounds by the reaction of **7**, **6**, and **8**, respectively, with one equivalent of 1-(bromoacetyl)pyrene in the presence of sodium methoxide in acetonitrile. The structures of all the new fluoroionophores were determined by FAB MS and NMR spectra.

**Figure 2:**
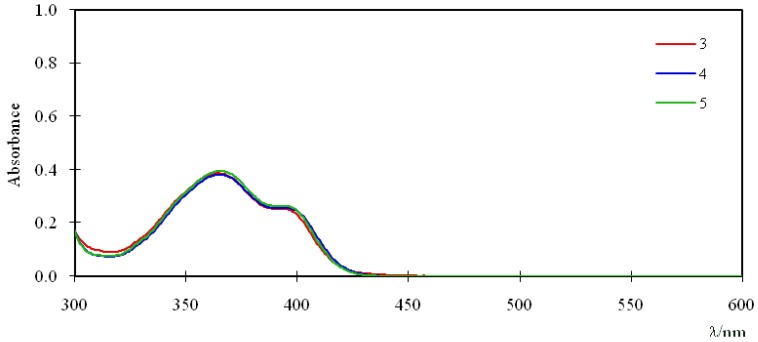
UV-Vis spectra of **3**, **4**, and **5 ** (10.0 μM in chloroform).

Figure 2 shows the UV-Vis spectra of 10.0 μM chloroform solutions of compounds **3**, **4**, and **5**. The difference of binding site X does not significantly modify the absorption spectra, characterized by an absorption maximum (λ_max_) at 366 nm and a shoulder at 393 nm.

**Figure 3:**
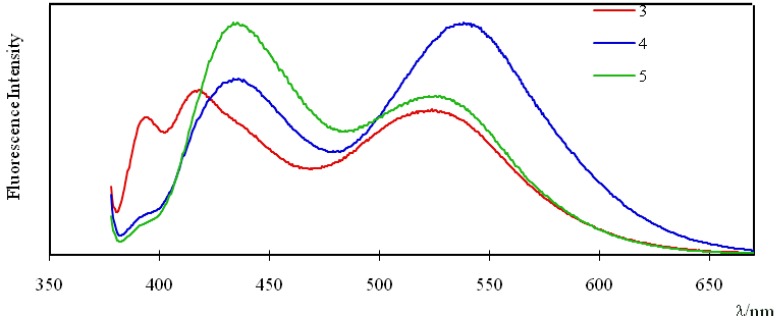
Fluorescence spectra of **3**, **4**, and **5** (1.0 μM in chloroform). Excitation wavelength: 360 nm.

Figure 3 shows the fluorescence spectra of 1.0 μM chloroform solutions of compounds **3**, **4**, and **5** under excitation at 360 nm. The pyrene monomer emission of compound **3** appeared at 380–440 nm, and those of compounds **4** and **5** appeared at 380–485 nm. On the other hand, the pyrene excimer emission of compounds **3**, **4**, and **5** appeared at different wavelengths: around 523 nm for **3**, 534 nm for **4**, and 527 nm for **5**. The fluorescence of pyrene in concentrated solutions shows dual emission bands: pyrene monomer emission at 370–425 nm, and excimer emission at around 480 nm [16,17]. The pyrene monomer and excimer emission bands of compounds **3**, **4**, and **5** were shifted to longer wavelengths than the corresponding emission bands of pyrene itself. These results can presumably be attributed to the effect of the electron-withdrawing carbonyl group [18,19,20,21].

It is noteworthy that the fluorescence intensity ratio (I_e_/I_m_) for compound **4** (1.32) is greater than that of compounds **3 **(0.88) and **5 **(0.69), where I_e_ and I_m_ are the fluorescence intensities of the pyrene excimer (at 523 nm for **3**, 534 nm for **4**, and 527 nm for **5**) and monomer (at 419 nm for **3** and 438 nm for **4** and **5**), respectively. These results indicate that the two pyrene units in compound **4** would be in closer proximity than those in compounds **3** and **5**.

**Figure 4:**
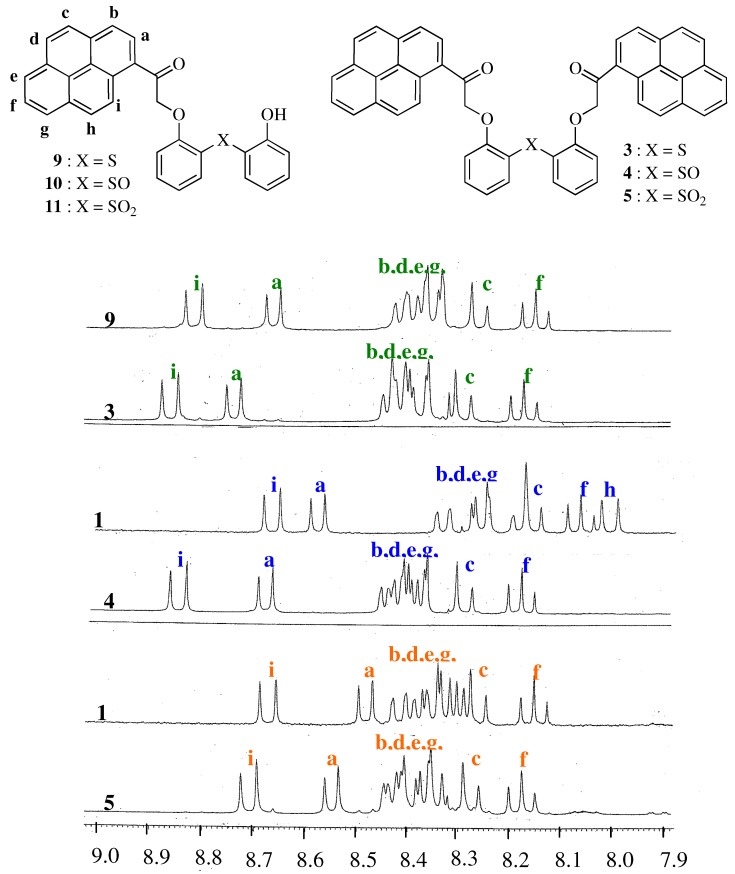
^1^H-NMR spectra of **3**, **4**, **5** (0.02 M in DMSO-*d*_6_) and **9**, **10**, **11** (0.04 M in DMSO-*d_6_*).

To examine the structures of compounds **3**, **4**, and **5** in detail, the ^1^H-NMR spectra of **3**, **4**, and **5** were compared with those of the corresponding compounds **9**, **10**, and **11**, which have one pyrene unit [22,23]. The ^1^H-NMR spectra of all compounds were examined in DMSO-*d_6_* by ^1^H-^1^H COSY, ^1^H-^13^C COSY, and HMBC spectroscopy. The spectra of the pyrene unit are shown in Figure 4. The chemical shifts (δ) of all compounds and the induced shifts (Δδ) are listed in Table 1.

**Table 1:** Chemical shift values (δ) of pyrene protons in compounds **3**–**5** and **9**–**11** and the induced shifts (Δδ) between the pyrene protons a–i in compounds **3**–**5**, with two pyrene units, and the corresponding pyrene protons in compounds **9**–**11**, with one pyrene unit.

Protons (δ: ppm)									
Compound	a	b	c	d	e	f	g	h	i
**9**	8.74	8.42	8.29	8.38	8.41	8.18	8.44	8.38	8.86
**3**	8.67	8.39	8.26	8.35	8.40	8.15	8.41	8.35	8.82
Δδ (**9–3**)	0.07	0.03	0.03	0.03	0.01	0.03	0.03	0.03	0.04
**10**	8.68	8.39	8.29	8.38	8.43	8.18	8.44	8.38	8.84
**4**	8.57	8.18	8.15	8.26	8.25	8.06	8.33	8.00	8.66
Δδ (**10–4**)	0.11	0.21	0.14	0.12	0.18	0.12	0.11	0.38	0.18
**11**	8.55	8.35	8.28	8.37	8.43	8.18	8.43	8.39	8.71
**5**	8.48	8.30	8.26	8.36	8.38	8.15	8.42	8.32	8.67
Δδ(**11–5**)	0.07	0.05	0.02	0.01	0.05	0.03	0.01	0.07	0.04

Table 1 shows that all the pyrene protons of **4** were shifted upfield compared to the corresponding protons of compound **10**. The Δδ (**10**–**4**) values are larger than the corresponding values of Δδ (**9**–**3**) and Δδ (**11**–**5**). These results suggest that both pyrene units in compound **4** are closer together than those in **3** and **5**, because it is well-established that π–π stacking interactions between two aromatic rings shield the protons due to the anisotropy of the ring current effect [23,24].

**Figure 5:**
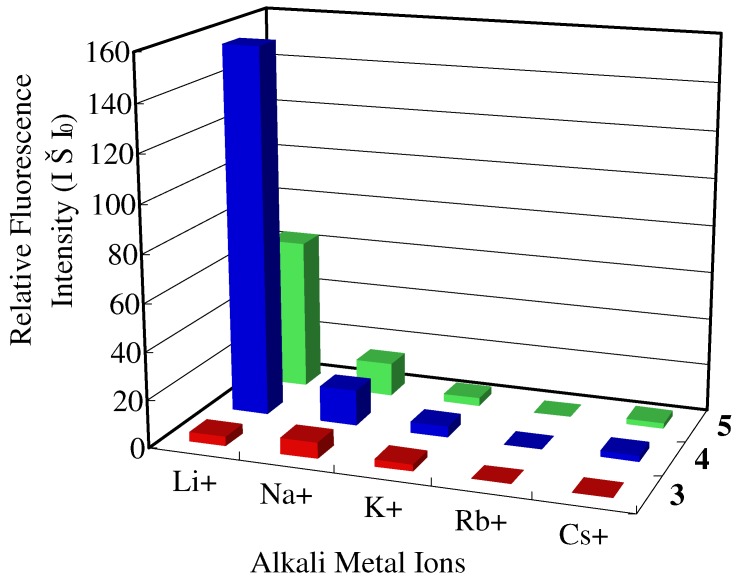
Effect of 300 μM alkali metal ions (Li^+^, Na^+^, K^+^, Rb^+^, Cs^+^) on the fluorescence spectra of compounds **3**, **4**, and **5** (1.0 μM) in chloroform-acetonitrile (97:3, v/v) at 419 nm for **3** and 438 nm for **4** and **5**. I_0_ and I are the fluorescence intensities of **3**, **4**, and **5** in the absence and presence, respectively, of alkali metal ions.

To examine the alkali metal ion binding abilities of compounds **3**, **4**, and **5**, we investigated the absorption and fluorescent spectral changes in chloroform-acetonitrile (97:3, v/v). No alkali metal ion-dependent changes in the UV-Vis spectra of **3**, **4**, and **5** were observed upon addition of 300 μM Li^+^, Na^+^, K^+^, Rb^+^, and Cs^+^ (all metal ions as perchlorate salts, except for Rb^+^ as thiocyanate salt to enhance solubility) to 1.0 μM **3**, **4**, and **5**. On the other hand, pyrene monomer emissions of **4** (at 438 nm), and **5** (at 438 nm) were enhanced by the addition of some alkali metal ions. Figure 5 shows the relative fluorescence intensity (I–I_0_), where I and I_0_ are the fluorescence intensity in the presence of 300 equiv. of each metal ion and the fluorescence intensity in the absence of metal ions, respectively. The addition of Li^+^ to solution **4**, which has a sulfinyl group, induced a remarkable intensity change. In contrast, a moderate change in the fluorescence of compound **5**, which has sulfonyl group, was observed with the addition of Li^+^. For **3**, which has a sulfur atom, no significant changes were observed. It is noteworthy that compounds **3**, **4**, and **5** interact slightly with Na^+^ compared with compound **2**[11]. The oxygen atom(s) of the SO and SO_2_ groups in compounds **4** and **5**, which bridge two phenyl groups, apparently have a crucial role in binding Li^+^, because even the compound **5** showed a moderate affinity to Li^+^. 

**Figure 6:**
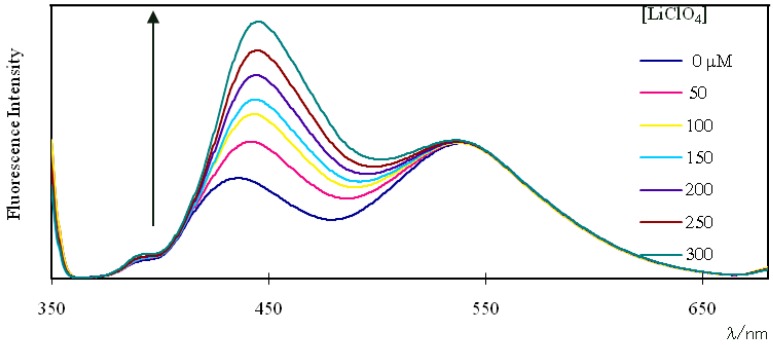
Changes in fluorescence spectra of a 1.0 μM solution of **4** in chloroform-acetonitrile (97:3, v/v) upon addition of LiClO_4_. Excitation wavelength: 360 nm.

Figure 6 shows the change in fluorescence of compound **4** with various Li^+^ concentrations under excitation at 360 nm. With the addition of Li^+^ in the range of 50–300 μM, the pyrene monomer emission increased dramatically, and the pyrene excimer emission changed slightly. Similar fluorescence changes were observed for compound **5** by the addition of Li^+^ (not shown).

**Figure 7:**
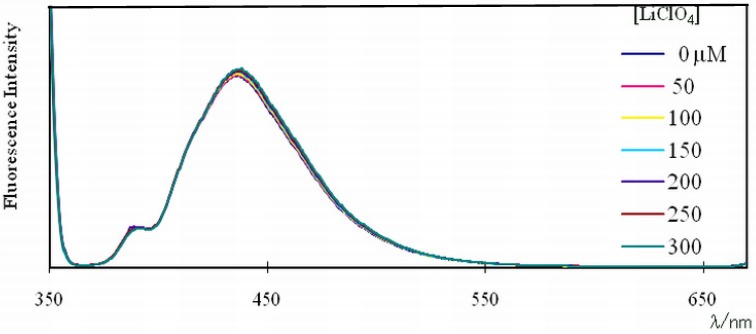
Changes in fluorescence spectra of a 1.0 μM solution of **10** in chloroform-acetonitrile (97:3, v/v) upon addition of LiClO_4_. Excitation wavelength: 360 nm.

As shown in Figure 7, the pyrene monomer emission of **10**, with one pyrene unit, was unchanged by the addition of Li^+^ in the range of 50–300 μM. This result clearly indicates that the increases in pyrene monomer emissions of compound **4** with increasing Li^+^ concentration were caused by interaction between the two pyrene units in **4**. Namely, these interactions in **4** weaken with increasing Li^+^ concentration.

**Figure 8:**
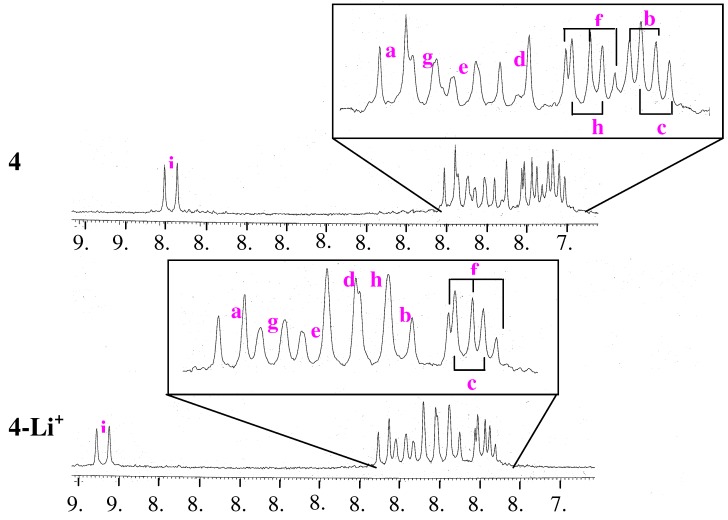
^1^H-NMR spectra of a 0.10 mM solution of **4** in CDCl_3_-CD_3_CN (97:3, v/v) in the absence (**4**) and presence (**4-Li^+^**) of Li^+^ (0.03 M).

To confirm the conformational changes in compound **4** caused by the addition of Li^+^, the chemical shifts of pyrene protons in compound **4** in the absence and presence of Li^+^ were examined in CDCl_3_-CD_3_CN (97:3, v/v) by ^1^H-NMR spectroscopy [7,25]. The spectra of the pyrene units in **4** in the absence (**4**) and presence (**4-Li^+^**) of Li^+^ are shown in Figure 8. The chemical shifts (δ) and induced shifts (Δδ) of pyrene protons are listed in Table 2. The signals for pyrene protons in **4-Li^+^** showed downfield shifts relative to the corresponding protons in **4**. These results indicate that the π–π stacking interaction between the two pyrene units in **4** is weakened by the addition of Li^+^.

**Table 2:** Chemical shifts values (δ) of pyrene protons in **4** and **4-Li^+^**, and the induced shifts (Δδ) between the pyrene protons in **4** and **4-Li^+^**.

Protons (δ: ppm)									
Compound	A	B	C	D	E	F	G	H	I
**4**	8.18	7.92	7.90	8.05	8.10	7.97	8.14	7.98	8.87
**4-Li^+^**	8.34	8.17	8.09	8.23	8.26	8.09	8.30	8.19	9.04
Δδ**(4-4-Li^+^)**	−0.16	−0.25	−0.19	−0.18	−0.16	−0.12	−0.16	−0.21	−0.17

The fluorophotometric and NMR spectroscopic studies described above indicate that the two pyrene units in compound **4** would be well separated in the presence of Li^+^ compared to their location in the absence of Li^+^. Therefore, the pyrene monomer emission is expected to increase and excimer emission is expected to decrease with increasing Li^+^ concentration. However, the pyrene excimer emission in **4** changed slightly with the addition of Li^+^, as shown in Figure 6. This is believed to be due to the small fluorescence intensity ratio (I_e_/I_m_) for compound **4** (1.32). In general, pyrene-functionalized fluoroionophores having a large fluorescence intensity ratio (I_e_/I_m_) before the addition of metal ions show a great decrease in pyrene excimer emission with increasing metal ion concentration. For example, two pyrene-functionalized calix[4]arenes having an (I_e_/I_m_) ratio of about 4.16, as reported by Jin et al., showed a great decrease in pyrene excimer emission with increasing Na^+^ concentration [26]. Even compound **2**, with an (I_e_/I_m_) ratio of 2.91, as we reported, showed a gradual decrease in pyrene excimer emission with increasing Na^+^ concentration [10].

**Figure 9:**
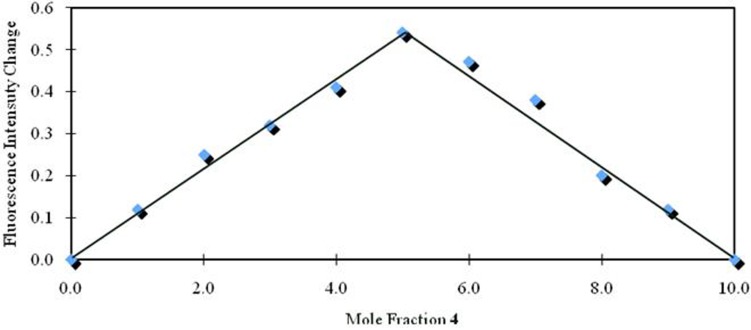
Job plot for the binding of Li^+^ with **4**.

The stoichiometry of compound **4** and Li^+^ was confirmed by a Job plot (Figure 9) using fluorescent titrations of 1.0 μM chloroform-acetonitrile (97:3, v/v) solutions of **4** with 1.0 μM chloroform-acetonitrile (97:3, v/v) solutions of lithium perchlorate. Consequently, the Job plot represented the formation of a 1:1 complex.

**Figure 10:**
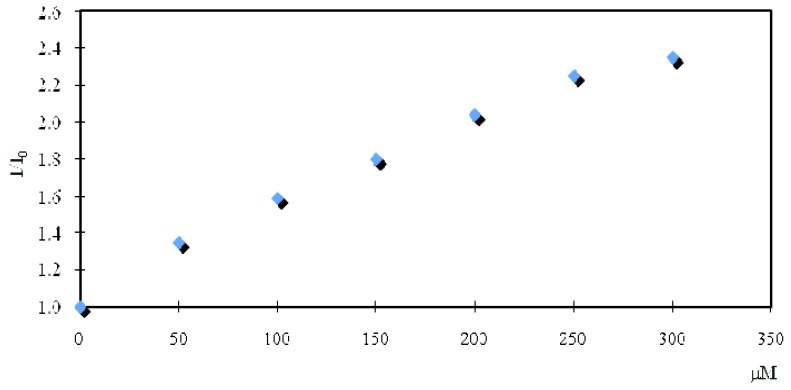
Relative fluorescence intensity (I/I_0_) of compound **4** (1 μM) in the absence and presence (0–300 μM) of Li^+^. The fluorescence intensities were monitored at 438 nm by excitation at 360 nm. I_0_ and I are the fluorescence intensity of **4** in the absence and presence, respectively, of Li^+^.

Figure 10 shows plots of the relative fluorescence intensity (I/I_0_) for **4** at 438 nm against the added Li^+^ concentration. The association constant of compound **4** for Li^+^ was calculated to be 1.14 × 10^3^ M^−1^ on the basis of the Benesi–Hildebrand method by plotting 1/(F–F_0_) against 1/[Li^+^], where F_0_ and F are the fluorescence intensities in the absence and presence, respectively, of Li^+^. By the same method, the association constant of compound **5** for Li^+^ was calculated to be 2.83 × 10^2^ M^−1^.

## 3. Experimental

### 3.1. General

All melting points were determined using a Hansen & Co., Ltd. MEL-TEMP and were not corrected. The fluorescence spectra were obtained by a Hitachi F-2500 fluorescence spectrophotometer. The ^1^H- and ^13^C-NMR spectra (at 300 MHz and 75 MHz, respectively) were measured using a JEOL-JNM-ECP300 spectrometer. The chemical shifts were measured in ppm downfield from the tetramethylsilane as an internal standard. The HRMS (FAB) spectra were measured by a JEOL JMS-SX102A using glycerol or 3-nitrobenzyl alcohol as the matrix. All chemicals for the synthesis of **3**–**11 **were commercially available and except for 1-(bromoacetyl)pyrene from Aldrich, were obtained from Tokyo Chemical Industry Co., Ltd. All reagents and solvents were used without further purification. Silica gel (70–230 mesh) came from Merck Ltd., Japan. Elemental analysis was performed by Mitsui Chemical Analysis & Consulting Service Inc., Japan.

### 3.2. General Procedure for Fluorescent Study

Stock chloroform solutions of compounds **3**–**5** and **10** (1.03 μM) and acetonitrile solutions of alkali metal salts LiClO_4_, NaClO_4_, KClO_4_, RbSCN, and CsClO_4_ (10 mM) were prepared by using spectroscopic-grade solvent (Wako Pure Chemical Industries, Ltd., Japan). Test solutions were prepared by mixing the stock solution (9.7 mL) of **3**–**5** and **10** and the alkali metal salt stock solution in increments of 0.00 mL, 0.05 mL, 0.10 mL, 0.15 mL, 0.20 mL, 0.25 mL, and 0.30 mL, after which the solution was diluted to 10.0 mL with acetonitrile.

### 3.3. General Procedure for the Preparation of 2,2´-Bis(1-pyrenylacetyloxy)diphenyl Compounds **3–5**

*2,2´-bis(1-Pyrenylacetyloxy)diphenyl sulfide* (**3**): A mixture of 2,2´-dihydroxydiphenyl sulfide (1.52 mmol, 0.33 g) [15], 1-(bromoacetyl)pyrene (3.10 mmol, 1.00 g) and potassium carbonate (4.56 mmol, 0.63 g) was refluxed in acetonitrile (300 mL) for 8 h. After evaporation of the solvent, the residue was dissolved in chloroform (300 mL). The chloroform solution was washed with water, and the solvent was evaporated. The residue was recrystallized from dichloromethane–hexane to afford compound **3**. Yield 83% (0.89 g) of yellow powder; mp 154–155 °C dec; ^1^H-NMR (DMSO-*d*_6_) δ 5.80 (s, 4H), 6.90 (td, *J* = 7.7 Hz, 1.1 Hz, 2H), 7.05 (dd, *J* = 7.7 Hz, 1.7 Hz, 2H), 7.06 (dd, *J* = 8.3 Hz, 1.1 Hz, 2H), 7.26 (td, *J* = 8.3 Hz, 1.7 Hz, 2H), 8.15 (t, *J* = 7.7 Hz, 2H), 8.26 (d, *J* = 9.1 Hz, 2H), 8.33–8.43 (m, 10H), 8.67 (d, *J* = 8.3 Hz, 2H), 8.82 (d, *J* = 9.3 Hz, 2H); ^13^C-NMR (DMSO-*d*_6_) δ 72.2, 112.6, 121.6, 122.1, 123.3, 124.0, 124.1, 124.3, 126.3, 126.7, 126.8, 127.0, 127.2, 128.4, 128.8, 128.9, 129.6, 129.7, 129.9, 130.5, 131.8, 133.7, 156.2, 198.6; HRMS (FAB): calcd for (M+H)/z: 703.1944. found (M+H)/z: 703.1943. Anal. calcd for C_48_H_30_O_4_S: C, 82.03; H, 4.30%; found: C, 81.87; H, 4.35%.

*2,2´-bis(1-Pyrenylacetyloxy)diphenyl sulfoxide* (**4**): This compound was prepared by the same procedure given for the synthesis of compound **3** except that 2,2´-dihydroxydiphenyl sulfoxide [15] (0.36 g) was used instead of 2,2´-dihydroxydiphenyl sulfide. Yield 67% (0.72 g) of yellow powder; mp 184–185 °C dec; ^1^H-NMR (CDCl_3_) δ 5.78 (d, *J* = 3.0 Hz, 4H), 7.10–7.16 (m, 4H), 7.46 (td, *J* = 8.5 Hz, 1.7 Hz, 2H), 7.58 (dd, *J* = 7.4 Hz, 1.7 Hz, 2H), 8.00 (d, *J* = 7.7 Hz, 2H), 8.06 (t, *J* = 7.7 Hz, 2H), 8.14–8.34 (m, 10H), 8.57 (d, *J* = 8.0 Hz, 2H), 8.66 (d, *J* = 9.6 Hz, 2H); ^13^C-NMR (DMSO-*d*_6_) δ; 72.2, 113.0, 121.6, 123.2, 123.9, 124.2, 126.0, 126.1, 126.6, 126.7, 126.9, 127.0, 128.1, 128.9, 129.6, 129.7, 129.8, 130.4, 132.4, 132.8, 133.7, 155.1, 197.6; HRMS (FAB): calcd for (M+H)/z: 719.1924. found (M+H)/z: 719.1895. Anal. calcd for C_48_H_30_O_5_S: C, 80.20; H, 4.21%. found: C, 79.98; H, 4.04%.

*2,2´-bis(1-Pyrenylacetyloxy)diphenyl sulfone* (**5**): This compound was prepared by the same procedure given for the synthesis of compound **3** except that 2,2´-dihydroxydiphenyl sulfone [15] (0.38 g) was used instead of 2,2´-dihydroxydiphenyl sulfide. Yield 72% (0.80 g) of yellow powder; mp 221–222 °C dec; ^1^H-NMR (CDCl_3_) δ 5.57 (s, 4H), 6.91 (t, *J* = 7.4 Hz, 2H), 7.24 (d, *J* = 8.0 Hz, 2H), 7.55 (td, *J* = 8.5 Hz, 1.7 Hz, 2H), 7.90 (dd, *J* = 8.0 Hz, 1.7 Hz, 2H), 8.15 (t, *J* = 7.7 Hz, 2H), 8.26 (d, *J* = 9.0 Hz, 2H), 8.29–8.43 (m, 10H), 8.48 (d, *J* = 8.0 Hz, 2H), 8.67 (d, *J* = 9.3 Hz, 2H); ^13^C-NMR (DMSO-*d*_6_) δ; 72.5, 114.2, 120.3, 123.3, 123.9, 124.0, 124.2, 126.3, 126.7, 126.8, 127.0, 127.1, 128.5, 128.6, 128.8, 129.6, 129.7, 129.8, 130.5, 133.6, 135.0, 155.6, 197.2; HRMS (FAB): calcd for (M+H)/z: 735.1842. found (M+H)/z: 735.1846. Anal. calcd for C_48_H_30_O_6_S: C, 78.46; H, 4.12%. found: C, 78.42; H, 4.12%.

### 3.4. General Procedure for the Preparation of 2-(1-Pyrenylacetyloxy)-2´-hydroxydiphenyl Compounds **9–11**

*2-(1-Pyrenylacetyloxy)-2´-hydroxydiphenyl sulfide* (**9**): A mixture of 2,2´-dihydroxydiphenyl sulfide (2.50 mmol, 0.54 g) [15] and sodium methoxide (2.91 mmol, 0.16 g) was refluxed in acetonitrile (80 mL) for 30 min to completely monodeprotonate the 2,2´-dihydroxydiphenyl sulfide. A solution of 1-(bromoacetyl)pyrene (1.00 g, 3.10 mmol) in acetonitrile (300 mL) was then added to the reaction mixture, followed by stirring at the reflux temperature for 6 h. The reaction mixture was neutralized with a few drops of acetic acid, and the solvent was removed. The residue was purified by recrystallization from chloroform to afford **9**. Yield 70% (0.80 g) of yellow powder; mp 154–155 °C dec; ^1^H-NMR (CDCl_3_) δ 5.85 (s, 2H), 6.79 (td, *J* = 7.8 Hz, 1.4, 1H), 6.80 (dd, *J* = 7.8 Hz, 1.7, 1H), 6.87 (td, *J* = 7.7 Hz, 1.1, 1H), 6.96 (dd, *J* = 8.0 Hz, 1.1, 1H), 7.07–7.24 (m, 4H), 8.18 (t, *J* = 7.7 Hz, 1H), 8.29 (d, *J* = 9.0 Hz, 1H), 8.32–8.45 (m, 5H), 8.74 (d, *J* = 8.3 Hz, 1H), 8.86 (d, *J* = 9.3 Hz, 1H), 9.89 (s, 1H); ^13^C-NMR (DMSO-*d*_6_) δ; 72.2, 79.2, 112.3, 115.7, 117.8, 119.9, 121.4, 123.4, 123.9, 124.0, 124.3, 126.3, 126.7, 126.9, 127.0, 127.2, 128.8 128.9, 129.0, 129.6, 129.8, 129.9, 130.6, 133.7, 134.1, 155.0, 157.2, 198.5; HRMS (FAB): calcd for (M+H)/z: 461.1212. found (M+H): 461.1206. Anal. calcd for C_30_H_20_O_3_S: C, 78.20; H, 4.38%. found: C, 77.90; H, 4.29%.

*2-(1-Pyrenylacetyloxy)-2´-hydroxydiphenyl sulfoxide* (**10**): This compound was prepared by the same procedure given for the synthesis of compound **9** except that 2,2´-dihydroxydiphenyl sulfoxide (0.59 g) [15] was used instead of 2,2´-dihydroxydiphenyl sulfide. Yield 25% (0.29 g) of yellow powder; mp 168–169 °C dec; ^1^H-NMR (CDCl_3_) δ 5.76 (d, *J* = 17.3 Hz, 1H), 5.87 (d, *J* = 17.3 Hz, 1H), 6.89 (td, *J* = 8.0 Hz, 0.84, 1H), 6.89 (dd, *J* = 7.4 Hz, 0.84, 1H), 7.16 (td, *J* = 7.4 Hz, 0.84, 1H), 7.20 (d, *J* = 7.7 Hz, 1H), 7.32 (td, *J* = 8.0 Hz, 1.7, 1H), 7.38 (dd, *J* = 7.7 Hz, 1.7, 1H), 7.48 (td, *J* = 7.4 Hz, 1.7, 1H), 7.55 (dd, *J* = 7.7 Hz, 1.7, 1H), 8.18 (t, *J* = 7.7 Hz, 1H), 8.29 (d, *J* = 9.1 Hz, 1H), 8.36–8.45 (m, 5H), 8.68 (d, *J* = 8.0 Hz, 1H), 8.84 (d, *J* = 9.4 Hz, 1H), 10.38 (s, 1H); ^13^C-NMR (DMSO-*d*_6_) δ; 72.3, 113.1, 116.2, 119.5, 121.6, 123.3, 124.0, 124.1, 124.3, 126.1, 126.3, 126.5, 126.8, 126.9, 127.0, 127.2, 128.5 128.9, 129.4, 129.8, 129.9, 130.5, 132.0, 132.3, 132.4, 133.8, 155.2, 155.4, 197.8; HRMS (FAB): calcd for (M+H)/z: 477.1161. found (M+H)/z: 477.1161. Anal. calcd for C_30_H_20_O_4_S: C, 75.61; H, 4.23%. Found: C, 75.31; H, 4.12%.

*2-(1-Pyrenylacetyloxy)-2´-hydroxydiphenyl sulfone* (**11**): This compound was prepared by the same procedure given for the synthesis of compound **9** except that 2,2´-dihydroxydiphenyl sulfone [15] (0.63 g) was used instead of 2,2´-dihydroxydiphenyl sulfide. Yield 27% (0.34 g) of yellow powder; mp 178–179 °C dec; ^1^H-NMR (300 MHz CDCl_3_) δ 5.62 (s, 2H), 6.71 (td, *J* = 8.0 Hz, 0.84, 1H), 6.84 (dd, *J* = 8.3 Hz, 0.84, 1H), 7.20 (t, *J* = 7.7 Hz, 1H), 7.21 (d, *J* = 8.5 Hz, 1H), 7.38 (td, *J* = 8.5 Hz, 1.7, 1H), 7.61 (td, *J* = 8.3 Hz, 1.7, 1H), 7.78 (dd, *J* = 8.0 Hz, 1.7, 1H), 8.06 (dd, *J* = 7.7 Hz, 1.7, 1H), 8.18 (t, *J* = 7.7 Hz, 1H), 8.30 (d, *J* = 9.0 Hz, 1H), 8.32–8.45 (m, 5H), 8.55 (d, *J* = 8.0 Hz, 1H), 8.71 (d, *J* = 9.4 Hz, 1H), 10.39 (s, 1H); ^13^C-NMR (DMSO-*d*_6_) δ; 72.3, 79.5, 114.0, 116.9, 118.0, 120.4, 123.3, 124.0, 124.1, 124.3, 126.1, 126.3, 126.8, 126.9, 127.1, 127.2, 128.5, 128.9, 129.7, 129.8, 129.9, 130.4, 130.5, 130.6, 133.7, 134.8, 135.0, 155.5, 155.7, 197.0; HRMS (FAB): calcd for (M+H)/z: 493.1110. found (M+H)/z: 493.1105. Anal. calcd for C_30_H_20_O_5_S: C, 73.16; H, 4.09%. found: C, 72.86; H, 4.08%.

## 4. Conclusions

We have synthesized new podand-type fluoroionophores by connecting two pyrene units with 2,2´-dihydroxydiphenyl sulfide, sulfoxide, and sulfone. The fluorescent change upon addition of alkali metal ions show that the difference in binding site affected metal ion selectivity and the fluoroionophore having sulfinyl group is highly selective for Li^+^ ion over other alkali metal ions. Although a number of fluoroionophores using calixarenes and crown ethers as ion recognition unit have been reported, these results demonstrated that a podand-type fluoroionophore with a non-cyclic binding site having sulfinyl group would be applicable as an effective Li^+^ ion fluorescence sensor. Along these lines, we are examining the synthesis of new fluoroionophore that can be able to monitor Li^+^ ion concentrations in aqueous samples.

## References

[1] Higuchi Y., Narita M., Niimi T., Ogawa N., Hamada F., Kumagai H., Iki N., Miyano S., Kabuto C. (2000). Fluorescent chemo-sensor for metal cations based on thiacalix[4]arenes modified with dansyl moieties at the lower rim. Tetrahedron.

[2] Callan J.F., de Silva A.P., Magri D.C. (2005). Luminescent sensors and switches in the early 21st century. Tetrahedron.

[3] Liu Y., Duan Z.-Y., Zhang H.-Y., Jiang X.-L., Han J.-R. (2005). Selective binding and inverse fluorescent behavior of magnesium ion by podand possessing plural imidazo[4,5-*f*]-1,10-phenanthroline groups and its Ru (II) complex. J. Org. Chem..

[4] Peng R., Wang F., Sha Y. (2007). Synthesis of 5-dialkyl(aryl)aminomethyl-8-hydroxyquinoline dansylates as selective fluorescent sensors for Fe^3+^. Molecules.

[5] Wang F., Peng R., Sha Y. (2008). Selective dendritic fluorescent sensors for Zn (II). Molecules.

[6] Wanichacheva N., Watpathomsub S., Lee V.S., Grudpan K. (2010). Synthesis of a novel fluorescent sensor bearing dansyl fluorophores for the highly selective detection of mercury (II) ions. Molecules.

[7] Jin T., Ichikawa K., Koyama T. (1992). A fluorescent calix[4]arene as a intramolecular excimer-forming Na+ sensor in nonaqueous solution. J. Chem. Soc. Chem. Commun..

[8] Nishizawa S., Watanabe M., Uchida T., Teramae N. (1999). Fluorescence ratio sensing of alkali metal ions based on control of the intramolecular exciplex formation. J. Chem. Soc. Perkin Trans..

[9] He H., Mortellaro M.A., Leiner M.J.P., Fraatz R.J., Tusa J.K. (2003). A fluorescent sensor with high selectivity and sensitivity for potassium in water. J. Am. Chem. Soc..

[10] Nishimura Y., Takemura T., Arai S. (2007). Effective fluorescent sensing of Na^+^ ion by calix[4]arene bearing pyrene and perylene based on energy transfer. ARKIVOC.

[11] Nishimura Y., Takemura T., Arai S. (2009). Effective Na^+^ fluorescent sensing by new podand-type receptor connecting two pyrene units and diphenyl ether. ARKIVOC.

[12] Wanichecheva N., Benco J.S., Lambert C.R., McGimpsey W.G. (2006). A highly selective bicyclic fluoroionophore for the detection of lithium ions. J. Photochem. Photobiol..

[13] Citterio D., Takeda J., Kosugi M., Hisamoto H., Sasaki S., Komatsu H., Suzuki K. (2007). pH-independent fluorescent chemosensor for highly selective lithium ion sensing. Anal. Chem..

[14] Rochat S., Grote Z., Severin K. (2009). Ruthenium-based metallacrown complex for the selective detection of lithium ions in water and serum by fluorescence spectroscopy. Org. Biomol. Chem..

[15] Gump W.S., Vitucci J.C. (1945). 2-Hydroxyphenyl sulfoxides and 2-hydroxyphenyl sulfones. J. Am. Chem. Soc..

[16] Guilbault G.G. (1990). Practical Fluorescence.

[17] Valeur B. (2006). Molecular Fluorescence Principles and Applications.

[18] Yuasa H., Miyagawa N., Izumi T., Nakatani M., Izumi M., Hashimoto H. (2004). Hinge sugar as a movable component of an excimer fluorescence sensor. Org. Lett..

[19] Kadirvel M., Bichenkova E.V., D’Emanuele A., Freeman S. (2006). Locked energy of axial to equatorial transformation monitored by exciplex and excimer fluorescence. Chem. Lett..

[20] Guilbault G.G. (1990). Practical Fluorescence.

[21] Valeur B. (2006). Molecular Fluorescence Principles and Applications.

[22] Venkataramana G., Sankararaman S. (2006). Synthesis and spectroscopic investigation of aggregation through cooperative π-π and C-H···O interactions in a novel pyrene octaaldehyde derivative. Org. Lett..

[23] Nandy R., Subramoni M., Varghese B., Sankararaman S. (2007). Intramolecular π-stacking interaction in a rigid molecular hinge substituted with 1-(pyrenylethynyl) units. J. Org. Chem..

[24] Shetty A.S., Zhang J., Moore J.S. (1996). Aromatic π-stacking in solution as revealed through the aggregation of phenylacetylene macrocycles. J. Am. Chem. Soc..

[25] Aoki I., Sakaki T., Shinkai S. (1992). A new metal sensory system based on intramolecular fluorescence quenching on the ionophoric calix[4]arene ring. J. Chem. Soc. Chem. Commun..

[26] Aoki I., Kawabata H., Nakashima K., Shinkai S. (1991). Fluorescent calix[4]arene which responds to solvent polarity and metal lons. J. Chem. Soc. Chem. Commun..

